# 3D Printing and Solvent Dissolution Recycling of Polylactide–Lunar Regolith Composites by Material Extrusion Approach

**DOI:** 10.3390/polym12081724

**Published:** 2020-07-31

**Authors:** Han Li, Wei Zhao, Xinhui Wu, Hong Tang, Qiushi Li, Jing Tan, Gong Wang

**Affiliations:** 1 CAS Key Laboratory of Space Manufacturing Technology, Technology and Engineering Center for Space Utilization, Chinese Academy of Sciences, Beijing 100094, China; lihan@csu.ac.cn (H.L.); xinhui.wu95s@outlook.com (X.W.); liqiushi17@csu.ac.cn (Q.L.); 2 University of Chinese Academy of Science, Beijing 100049, China; tanghong@vip.gyig.ac.cn; 3 Institute of Geochemistry, Chinese Academy of Sciences, Guiyang 550081, China; 4 School of Mechanical Engineering, Jiangsu University, Zhenjiang 212013, China; tanjing0916@163.com

**Keywords:** 3D printing, in situ resource utilization, recycling, lunar regolith

## Abstract

The in situ resource utilization of lunar regolith is of great significance for the development of planetary materials science and space manufacturing. The material extrusion deposition approach provides an advanced method for fabricating polylactide/lunar regolith simulant (PLA/CLRS-1) components. This work aims to fabricate 3D printed PLA–lunar regolith simulant (5 and 10 wt.%) components using the material extrusion 3D printing approach, and realize their solvent dissolution recycling process. The influence of the lunar regolith simulant on the mechanical and thermal properties of the 3D printed PLA/CLRS-1 composites is systematically studied. The microstructure of 3D printed PLA/CLRS-1 parts was investigated by scanning electron microscopy (SEM) and X-ray computed tomography (XCT) analysis. The results showed that the lunar regolith simulant can be fabricated and combined with a PLA matrix utilizing a 3D printing process, only slightly influencing the mechanical performance of printed specimens. Moreover, the crystallization process of PLA is obviously accelerated by the addition of CLRS-1 because of heterogeneous nucleation. Additionally, by using gel permeation chromatography (GPC) and attenuated total reflectance Fourier transform infrared (ATR-FTIR) characterization, it is found that the 3D printing and recycling processes have a negligible influence on the chemical structure and molecular weight of the PLA/CLRS-1 composites. As a breakthrough, we successfully utilize the lunar regolith simulant to print components with satisfactory mechanical properties and confirm the feasibility of recycling and reusing 3D printed PLA/CLRS-1 components via the solvent dissolution recycling approach.

## 1. Introduction

Returning humans to the Moon has attracted the attention of the world’s leading space powers. Both China and the United States have announced their intention to conduct manned lunar exploration in the near future. Due to the long distance between Earth and the Moon, and the high transportation costs, in situ resource utilization (ISRU) has been recognized as a cost-effective and efficient approach for long-stay manned operations [1,2,3]. Lunar regolith, which covers the surface of the Moon, consists of fine particles with particle sizes smaller than 1 mm. Lunar regolith is thought to have been produced from the combined action of micrometeorites, charging particles and radiation. Previous works have focused on sintering lunar regolith as potential construction materials, such as bricks, stabilized landing pads, roads, etc. [4,5,6].

In recent years, due to advances in 3D printing, it has become possible to fabricate multifunctioning parts with complex shapes during Moon exploration missions [7,8,9,10]. Two kinds of materials can be fabricated by 3D printing on the Moon. One type is materials carried by humans, while the other is related to resources constructed from lunar regolith, water–ice, etc. [11,12,13]. Blending lunar regolith with additives is a popular method to provide filaments for 3D printing [14,15,16]. The European Space Agency (ESA) [17] has reported an inkjet printing method to fabricate 3D shaped stone specimens by blending lunar regolith with a chlorine-based liquid. Additionally, Shah et al. [18] presented the extrusion-based 3D printing of regolith simulant inks, which were composed of regolith simulants, evaporants, surfactants and plasticizer solvents. Wang et al. [19,20] prepared sintered components by the digital light processing and sintering of a mixture of photopolymers, photoinitiators and lunar regolith simulants.

Although the concept of 3D printing with blended lunar regolith mixtures has been proven, there are still many challenges that will likely be faced by future lunar missions. First, additives are disposable, and cannot be reused in a resource-starved environment. Second, a number of complex and high energy-consumption devices, such as a sintering furnace, are required for the 3D printing process [21,22,23]. Lastly, the mechanical properties of lunar regolith-containing 3D printed parts should be improved. The traditional material deposition approach has shown the ability to fabricate complex thermoplastic polymer parts on demand on the International Space Station (ISS) [24,25]. In the material deposition approach, components are fabricated by melting and extruding polymer-based filament through a nozzle on pre-determined routes. After one layer is completed, the print bed is moved down before the next layer begins. The process does not need much power and is easily adopted to print parts for different fields [26]. This approach may also be suitable for manned Moon exploration. In addition, the reutilization of thermoplastic polymers, which are important for deep-space exploration, are currently under experimental verification on the ISS [27]. On Earth, the demand for the reutilization of 3D printed parts is also growing [28,29]. However, the melt extrusion process may cause the degradation of thermoplastic polymers. The more cycles, the more serious the degradation. Moreover, although numerous studies have been conducted regarding 3D printing using lunar regolith, previous works have not addressed the influence of lunar regolith on material-deposition processing and 3D printed thermoplastic composites.

To address this paucity of studies, in this work, we developed a material extrusion process for fabricating specimens with soluble, biocompatible polylactide (PLA) and lunar regolith simulant, followed by a dissolution–recycling process. Material filled with rich lunar regolith resources greatly improved the usage efficiency of PLA polymer materials on the lunar surface. The influence of the lunar regolith on the mechanical and thermal properties was also characterized. The enhanced mechanism of lunar regolith on PLA was also investigated by scanning electronic microscopy (SEM) and X-ray micro-computed tomography (XCT).

## 2. Experimental Section

### 2.1. Raw Materials and Filament Preparation

PLA pellets (trade name 4032D) with an average molecular weight (*M*_w_) of 2.49 × 10^5^ g/mol were purchased from NatureWorks^®^ LLC (Minnetonka, MN, USA). The lunar regolith simulant CLRS-1, supplied by the Institute of Geochemistry, Chinese Academy of Sciences, is an analogue of an Apollo 11 lunar soil sample [30,31,32]. The median particle diameter of CFLRS-1 is about 80–110 μm. Before bending with PLA, the lunar regolith simulants were milled into finer powder by a planetary ball mill. Prior to use, the PLA and CLRS-1 simulants were dried for 4 h at 80 °C. Before adding them into a volumetric single-screw feeder, the PLA and CLRS-1 simulants were mixed by a high-speed mixer at a stirring speed of 300 rpm/min for 10 min.

PLA and PLA/CLRS-1 composite filaments were prepared via melt blending using a Process 11 parallel twin-screw extruder (Thermo Scientific^TM^ HAAKE^TM^, Karlsruhe, Germany). The twin-screw contained three mixing sections consisting of staggered kneading disks (5KB30°/3KB60°/4KB90°; 6KB60°; 4KB60°/8KB90°) in order to promote desperation. The extruder was equipped with a volumetric single-screw feeder. The speeds of the twin-screw and single-screw feeder were set at 90 and 5 rpm, respectively. The processing temperatures from hopper to die ranged from 160 to 195 °C. The amount of CLRS-1 in PLA/CLRS-1 composites was controlled to 5 and 10 wt.%. Filaments were collected using a rolling-up device with an air-cooling device in order to obtain a filament diameter of approximately 1.75 ± 0.05 mm.

### 2.2. Fabrication of 3D Printed PLA/CLRS-1 Specimens

The tensile and bending specimens were designed by CATIA V5 according to ISO 527:2:2012 [33] and ISO 178:2010 [34], and printed by a FUNMAT HT FDM 3D printer (INTAMSYS, Shanghai, China). All specimens were printed with identical printing parameters, as shown in Table 1. The nozzle temperature was set at 200 °C to ensure the bonding strength of filaments and reduce the degradation of the PLA matrix. The other parameters were determined by an optimization process using neat PLA.

### 2.3. Recycle Experiment of 3D Printed PLA/CLRS-1 Specimens

The recycling process was performed on an oil bath equipped with a Soxhlet extractor. The gear shape of 3D printed PLA/CLRS-1 specimens was placed into an extraction thimble. A microporous membrane filter with a pore size of 0.22 μm was put into the bottom of the extraction thimble. During the recycling process, the solvent was tetrahydrofuran (THF), and the temperature of the oil bath was set at 80 °C. A few pieces of broken porcelain were put into the round bottom flask to prevent bumping. The recycling process lasted for about 2 h. After the recycling process, the lunar regolith simulant was left on the microporous membrane filter, and the reclaimed PLA powder and solvent were separated via distillation under reduced pressure.

### 2.4. Characterization

Tensile and bending tests were performed on an Instron 5965 universal testing machine (INSTRON Co., Ltd., Norwood, MA, USA) with a 5kN load capacity. The tensile and bending strength of 3D printed specimens were tested according to ISO 527:2:2012 [33] and ISO 178:2010 [34], respectively. A three-point bending mode was used to test the samples. The testing speed was set to 1 mm/min. At least five specimens of each sample were tested, and testing data were averaged.

Thermogravimetric analysis (TGA) was performed on a TGA/DSC-1 thermogravimetric analyzer (Mettler-Toledo Ltd., Greifensee, Switzerland). The samples were heated with a heating rate of 10 °C /min from 40 to 800 °C under nitrogen atmosphere. Differential scanning calorimetry (DSC) was carried out on a DSC 250 differential scanning calorimeter (TA Co., Ltd., New Castle, DE, USA) performing the following thermal cycle under a nitrogen atmosphere. The heating and cooling rate was set as 10 °C /min, and the temperature ranged from 30 to 200 °C. All samples were cut from the printed parts and tested without removing their heat history. Crystallinity was determined by the following equation:

$$\chi_{c}\left(\%\right)=\frac{∆H_{m}-∆H_{cc}}{\left(1-\varphi \right)∆H_{0}}\times 100\%$$

where ∆*H*_m_, ∆*H*_cc_ is the melting enthalpy and cold crystallization enthalpy of the sample analyzed (J/g), *∆H*_0_ is a reference value that represents the melting enthalpy of a 100% crystalline polymer (for a 100% crystalline PLA is 93 J/g), and *φ* is the CLRS-1 weight percentage.

Scanning electron microscopy (SEM) and elemental mapping (EDS) were performed using a Quanta 650 FEG field emission scanning electron microscope (Thermo Fisher Scientific Co., Ltd., Waltham, MA, USA) at an accelerating voltage of 10 kV, equipped with an energy dispersive X-ray spectrometer. The observed samples were obtained from the brittle fracture of tensile specimens under liquid nitrogen. Prior to observation, the testing samples were coated with a gold film for 60 s. Three-dimensional reconstruction imaging of the 3D printed flexural specimens was carried out by a Skyscan 1172 X-ray μ-CT system (Bruker Corp., Billerica, MA, USA). For printed specimens, the scanning pixel resolution was around 2.8 μm, and the image acquisition region was approximately 5 mm. After image acquisition, the 2D images were processed further to calculate the porosity, pore distribution and pore size of the specimens with Skyscan CTAn software (Bruker Corp., MA, USA).

Gel permeation chromatography (GPC) analysis was carried out using a Waters 1515 solution chromatograph instrument (Waters, Milford, MA, USA) with a 15–30 μL sample injection volume. In the experiment, PLA samples of about 5 mg were dissolved in 1 mL of tetrahydrofuran. Fourier transform infrared spectrometry was recorded on a Nicolet iS10 FTIR spectrometer (Thermo Fisher Scientific Co., Ltd., Waltham, MA, USA) in attenuated total reflectance mode. The PLA samples before and after the recycling process were analyzed in the 600–2000 cm^−1^ range with a 4 cm^−1^ resolution.

## 3. Results and Discussion 

### 3.1. Mechanical Behavior

**Figure 1:**
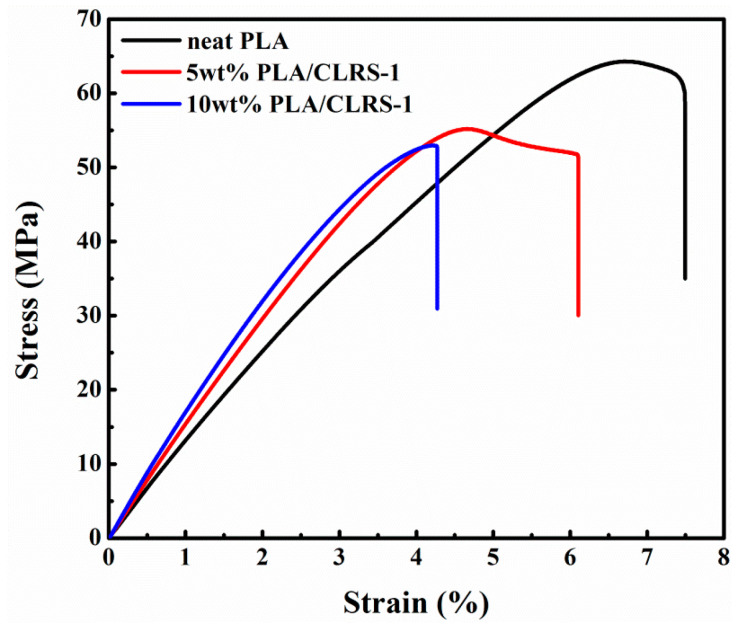
Typical tensile stress–strain curves of 3D printed neat polylactide (PLA) and polylactide/lunar regolith simulant (PLA/CLRS-1) samples for different contents.

Figure 1 presents the typical stress–strain curves of the 3D printed PLA and PLA/CLRS-1 composites, where the tensile strength and the amount of elongation at break are summarized in Table 2. Before fracturing, all 3D printed specimens underwent linear elastic deformation ranging from 4% to 8%, but thereafter, the specimens behaved differently. The 3D printed specimens made of PLA and PLA/CLRS-1 composites with 5 wt.% CLRS-1 yielded after the maximum stress (64.3 and 55.2 MPa) before fracturing. Meanwhile, the PLA/CLRS-1 composite specimens containing 10 wt.% CLRS-1 exhibited little plastic deformation prior to fracturing, although brittle fracture patterns were observed. These results showed that the addition and content of CLRS-1 had a great influence on the properties and fracture modes of the printed specimens.

The tensile properties of the printed PLA/CLRS-1 composite specimens were compared to those of the neat PLA in terms of the relative difference (% R.D.). The ultimate tensile strength of the 5 wt.% and 10 wt.% CLRS-1 samples was reduced by 14.2% and 17.6%, respectively, compared to that of neat PLA. Obviously, all printed PLA/CLRS-1 specimens presented a higher elasticity modulus compared to that of neat PLA (Figure 1). The higher elastic modulus was achieved in printed PLA/CLRS-1 composites, which agrees with the results of Paspali’s work regarding the incorporation of nanoclay into PLA [35]. The amount of elongation at break of the PLA/CLRS-1 composites also presented similar characteristics as seen for the tensile strength.

**Figure 2:**
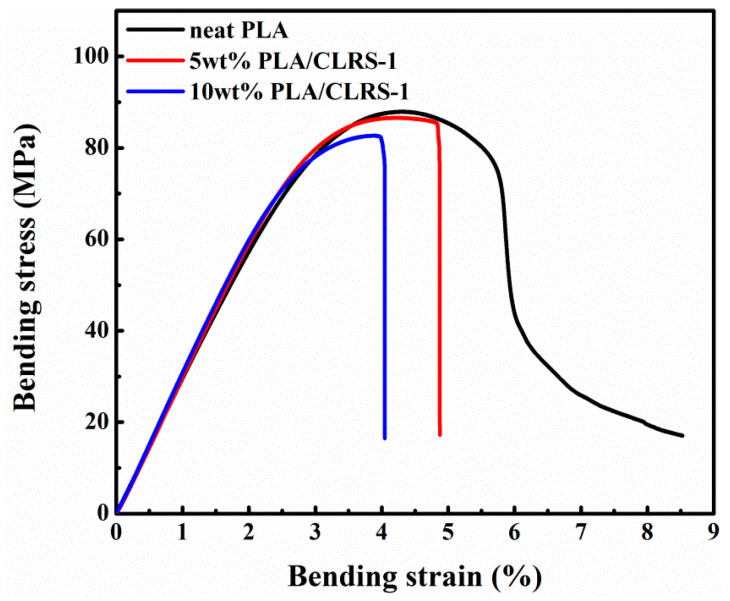
Typical bending stress–strain curves of 3D printed neat PLA and PLA/CLRS-1 samples for different contents.

Figure 2 presents the typical stress–strain curves of the printed specimens, obtained from the three-point bending tests, where the detailed data are summarized in Table 3. All printed specimens exhibited linear elastic deformation before the yield point. The neat PLA did not break during the test, and showed good toughness, while the 5 wt.% and 10 wt.% PLA/CLRS-1 composites fractured after yielding. The trends seen in bending test results were similar to those from the tensile tests, such as an increased modulus, and reduced strength and elongation. With an increased content of CLRS-1, the printed PLA/CLRS-1 composites could also achieve a high bending strength, as well as the neat PLA. Moreover, the bending modulus of the printed PLA/CLRS-1 composites increased due to the incorporation of the CLRS-1. This phenomenon indicated the good dispersity of CLRS-1 in the PLA matrix, and the CLRS-1 simulant in PLA. In comparison with previous research, 10 wt.% of CLRS-1 into PLA only shows a 7.39% reduction in the bending strength of printed composites, compared to a 21.1% reduction in PLA composites with 5 wt.% nanoclay [35]. This phenomenon lays the foundation for the use of PLA/CLRS-1 composites. In the following sections, further studies are performed to understand the mechanical mechanism of lunar regolith simulant in 3D printed PLA.

### 3.2. Mechanism of the Mechanical Behavior of PLA/CLRS-1 Composite Materials

**Figure 3:**
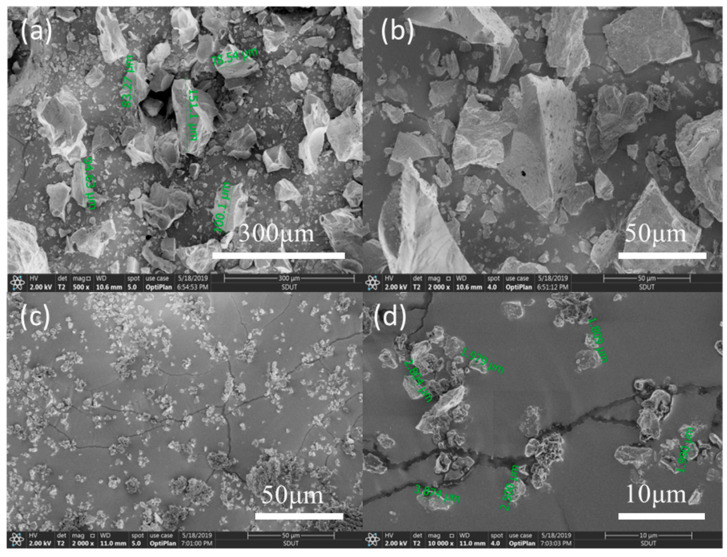
SEM micrographs of the lunar regolith simulants powder. (**a**,**b**) before the ball mill. (**c**,**d**) after the ball mill.

The morphologies of CLRS-1 before and after processing are shown in Figure 3. Pristine lunar regolith, before ball milling, exhibited irregular jagged contours, with a particle size of approximately 100 μm. Meanwhile, the milled CLRS-1 particles were smoother, with fewer contours, and the average particle size was ~2.5 μm. These images demonstrated that the ball milling process could significantly reduce the size of the lunar regolith powder, so that it could be mixed with the PLA better.

**Figure 4:**
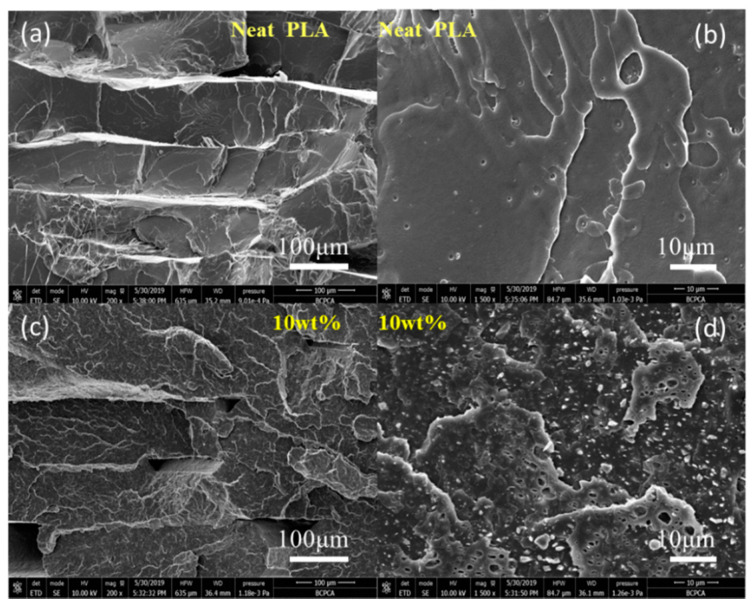
SEM micrographs of the cross-section of 3D printed tensile specimens after wetting-off in liquid nitrogen. (**a**,**b**) Neat PLA with different magnification. (**c**,**d**) PLA/10 wt% CLRS-1 composite with different magnification.

Figure 4 displays SEM images of a fractured section of the printed, neat PLA, and the PLA/CLRS-1 composite with a loading of 10 wt.% CLRS-1. As shown in Figure 4a,c, a regular, stacked, layered structure and smooth surfaces were present for both the neat PLA and PLA/CLRS-1 composites; these topologies are associated with the brittle fracture mode of the printed specimens. There were fewer small holes in the cross section of neat PLA (Figure 4b), due to the degradation of the PLA molecules during the printing process. As depicted in Figure 4c, there were more obvious triangular holes between adjacent layers, and more cracks on the surface of the fractured section of the PLA/CLRS-1 composite. This phenomenon may be related to the increased melt viscosity and crack deflection caused by the addition of the lunar regolith simulant. Moreover, the increased porosity and crack deflection could respond to the reduced of strength. From Figure 4d, it can be seen that the lunar regolith simulant particles were uniformly distributed on the surface. 

**Figure 5:**
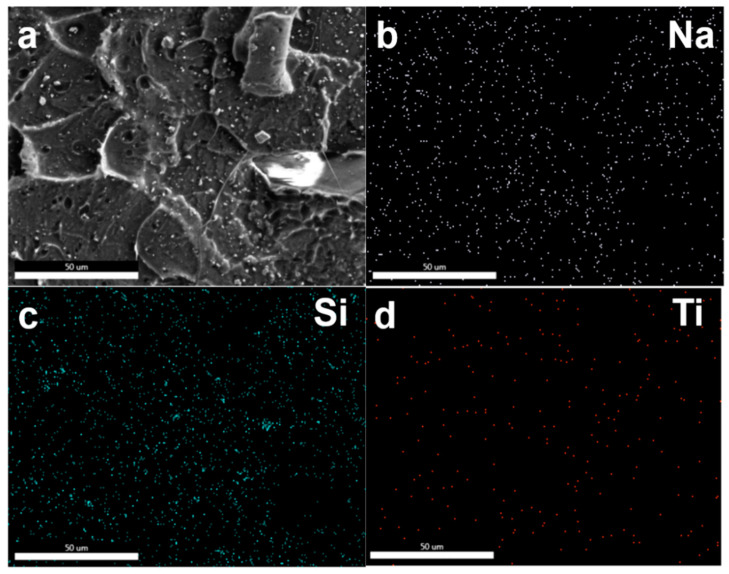
EDS mapping of lunar regolith dispersed in the PLA matrix. (**a**) SEM image of cross sections from PLA/CLRS-1 composites (**b**) Na; (**c**) Si; (**d**) Ti.

It is well known that lunar regolith consists of several kinds of oxides, such as silicates, metal oxides, and so on. To further demonstrate the dispersity of the multi-oxides in the polymer matrix, the lunar regolith present in the PLA matrix was analyzed by energy dispersive X-ray spectroscopy (EDS). As seen in the EDS mapping images (Figure 5), typical Na, Si and Ti elements were uniformly dispersed on the fractured surface of the PLA matrix.

**Figure 6:**
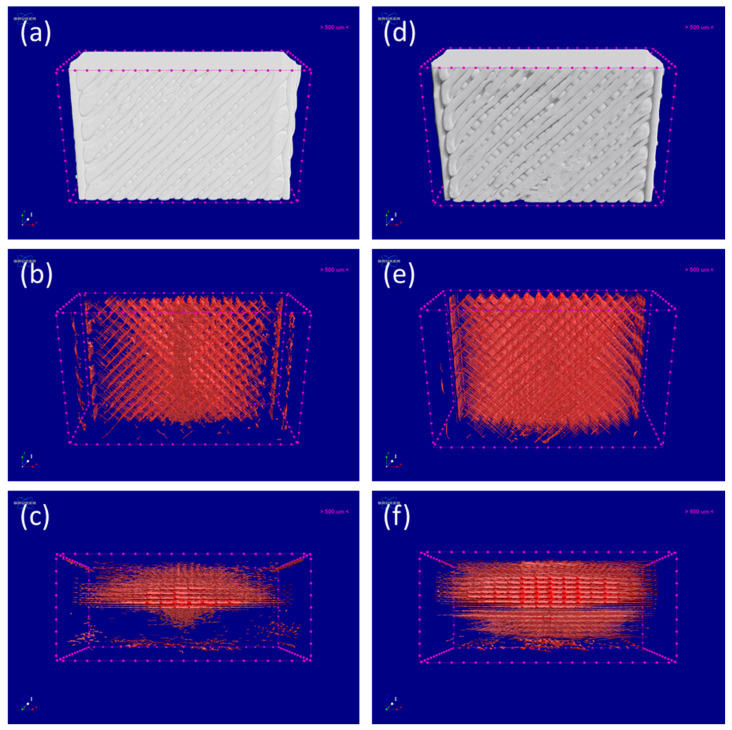
X-ray computed tomography (XCT) scanning of 3D printed PLA and PLA/CLRS-1 composites. Left (**a**–**c**): PLA; Right (**d**–**f**): PLA/10% CLRS-1. (**a**,**d**) present the schematic diagram of XCT scanning sample. (**b**,**c**,**e**,**f**) illustrate the 3D image from different angles. The Z-axis is the printing orientation, Y-axis is building orientation, and the deep orange parts are pores.

**Figure 7:**
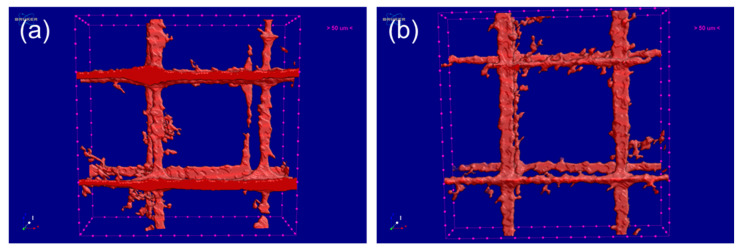
XCT scanning of pore size of 3D printed (**a**) PLA and (**b**) 10 wt% PLA/CLRS-1 composites.

To further study the microstructures of the printed PLA/CLRS-1 samples, an XCT analysis was used to determine their porosities, pore distributions and pore sizes. In recent years, XCT has played an increasingly important role in the nondestructive testing of 3D printed parts. Figure 6 and Figure 7, respectively, display the 3D reconstructed pore distributions of the PLA and PLA/CLRS-1 samples. On the outside surfaces of the printed specimens (Figure 6a,b), it can be seen that with the addition of lunar regolith, the gaps between adjacent extruded filaments have been enlarged. In terms of the 3D reconstructed pore distributions of the printed specimens, a regular crossed inter-connective porous structure was formed in all the printed specimens, which revealed the 3D printing path. Moreover, it can be concluded that the pore distribution of both printed specimens was inhomogeneous, on account of the temperature gradient of the build chamber. This is because the initial printed layer, which was closer to the print bed, experienced higher temperatures, which increased the flow of the extruded melt and reduced the gaps between adjacent filaments and layers. The crossed inter-connective porous structure and inhomogeneous pore distribution was also consistent with our previous work on 3D printed PEEK and CF/PEEK parts [9].

From Figure 6c,f, it can be seen that more pores and gaps appeared in the 3D images of the PLA/CLRS-1 composites, relative to the neat PLA samples. Statistically, the porosity of the 10wt.% PLA/CLRS-1 composite was 5.20%, while the porosity of the neat PLA sample was 4.18%. The porosity of the 3D printed PLA and PLA/CLRS-1 composite shows little difference compared to previous studies [36]. Based on the results of the porosity and pore distribution study, we investigated the pore sizes in the inner regions of the printed, neat PLA sample and 10 wt.% PLA/CLRS-1 composite further, as shown in Figure 7. The pores were mutually and perpendicularly orientated, forming a rectangular structure, whose dimensions are given in Table 4. As for the neat PLA sample, the length and width of the rectangular structure were 0.38 mm and 0.37 mm, respectively, while those of the 10 wt.% PLA/CLRS-1 composites were 0.33 mm and 0.39 mm, respectively. We found that the length and widths were all close to 0.4 mm, which was the filament diameter produced by the nozzle. More interestingly, the pore spacings of the neat PLA sample and the 10 wt.% PLA/CLRS-1 composite were 0.09 mm and 0.08 mm, respectively, which were close to the 0.1-mm layer thickness of the printed specimens. This could be related to interlayer overlapping and silting during the printing process. Therefore, the 10wt.% PLA/CLRS-1 composite specimens were thinner than the neat PLA specimens. This could be associated with the increased melt viscosity caused by the addition of lunar regolith, and interface effects between the PLA and lunar regolith. 

In conclusion, the microstructure and pore analysis results are in accordance with the decreased mechanical properties of the 3D printed PLA/CLRS-1 composites, where the increased porosity may be responsible for the lower bonding strength of the PLA/CLRS-1 composites.

### 3.3. Thermal Properties

**Figure 8:**
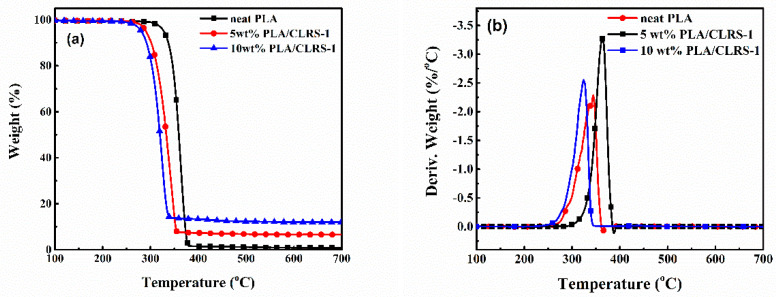
The thermogravimetric curves (**a**) and differential thermogravimetric curves (**b**) of 3D printed neat PLA and PLA/CLRS-1 composite specimens.

To study the influence of the lunar regolith on the thermal properties of PLA, TGA and DSC analyses were performed on the 3D printed PLA and PLA/CLRS-1 specimens. Figure 8 shows the TGA and DTG curves of the 3D printed PLA and PLA/CLRS-1 composites, where detailed data, such as the temperatures at which 5% weight losses occurred (*T*_5%_), the maximum decomposition temperature (*T*_max_) and residual char of 700 °C, are listed in Table 5. The *T*_5%_ values of the PLA/CLRS-1 composites decreased from 328 °C to 290 °C with the addition of 5 wt.% CLRS-1. The further addition of CLRS-1 up to 10 wt.% led to a lower *T*_5%_ of 279 °C, which was associated with the catalysis effect of the metal oxide and phosphate in the CLRS-1 lunar regolith simulant [37,38,39]. With the addition of CLRS-1, the *T*_max_ values of the PLA/CLRS-1 composites also shifted to low temperatures. Moreover, the residual char of the PLA/CLRS-1 composites at 700 °C increased with the increased content of CLRS-1. This enhancement of char formation was due to the hindering ability of CLRS-1, which prevented the polymer matrix from degrading further at higher temperatures. The CLRS-1 component in the PLA/CLRS-1 composites mainly acted as barrier to hinder the transport of pyrolysis gaseous products, thus affecting the degradation process [40].

**Figure 9:**
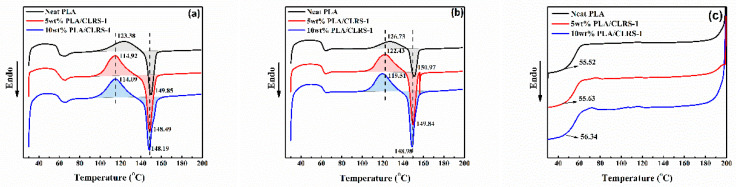
Differential scanning calorimetry thermogram of (**a**) the first heating, (**b**) the second heating, (**c**) the first cooling of 3D printed neat PLA and PLA/CLRS-1 composite specimens.

As a semi-crystalline polymer, the crystallinity of PLA has a significant influence on the mechanical properties. Figure 9a presents the DSC curves of the 3D printed PLA and PLA/CLRS-1 composites, whereas the detailed data are summarized in Table 5. Among those, crystallinity was calculated according to equation (1) and both enthalpies are integrated from the first heating curves to reflect the crystallization process of printed parts. The first heating curve reflects the thermal performance of the neat PLA and PLA/CLRS-1 composites during the 3D printing process, and the second heating scan reflects the intrinsic thermal properties of them. The addition of CLRS-1 has a slight influence on *T*_g_ and the crystallinity of the PLA matrix. However, with respect to the cold crystallinity behavior, the results show that the crystallization temperature is reduced with the content of the lunar soil simulant (from 123.38 to 114.09 °C) and the corresponding enthalpy is increased (from 14.76 to 21.18 J/g), which exhibits narrower and sharper peak on DSC curves. This phenomenon mainly contributes to the heterogeneous nucleation effect of CLRS-1 microparticles uniformly dispersed in the PLA matrix. Because CLRS-1 restricted the chain mobility of PLA, spherocrystal and lamellar crystal grow more easily under a relatively low temperature. Moreover, Figure 9b shows the intrinsic heat flow process of PLA/CLRS-1. Compared to neat PLA, the second heating curve indicates that the 3D printing process and CLRS-1 both improve the cold crystallization process, while they do not influence the whole degree of crystallinity of the final product (all crystallinities are lower than 4%).

### 3.4. Recycling Process

ISRU is regarded as a key technology to carry out manned extraterrestrial and deep-space exploration. With the ISRU approach, new tools and supplies for scientific experiments can be made from the bio-packing of materials and printed parts. There are two main methods used to recycle polymer materials, including solvent dissolution recycling and grinding cycle processing. Along these lines, NASA has deployed a recycling machine on the ISS, which has been used to test recycling processes under microgravity. The recycling machine, named the ‘Refabricator’, can break waste plastic into pieces and turn it into feedstock for the printer to create new items by the melt extrusion method [27].

**Figure 10:**
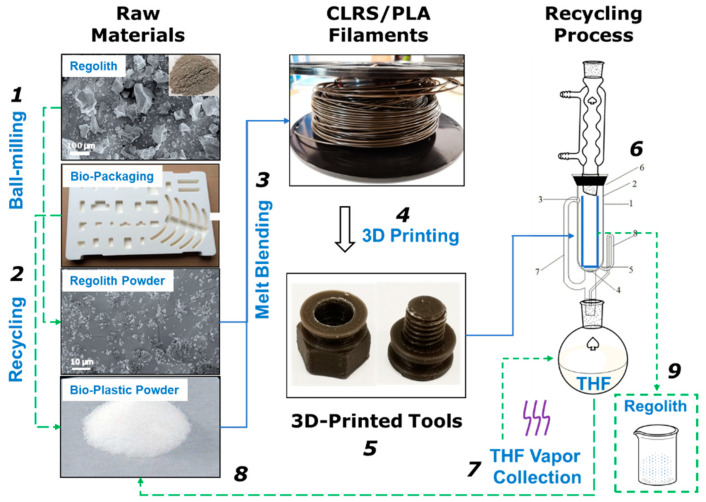
Schematic diagram of the fabrication and recycling of the component, lunar regolith ball milling (**1**), PLA recycling (**2**), raw material regolith and PLA melt blending, extrusion (**3**), 3D printing (**4**), final 3D printed tools (**5**), recycling process (**6**), tetrahydrofuran (THF) vapor collection (**7**), recycled PLA (**8**), recycled regolith (**9**).

Here, we adopted the fat extractor reflux method, which is a solvent dissolution method, to recycle PLA and lunar regolith from 3D printed components. Figure 10 shows the fabrication and recycling processes of the 3D printed parts. The PLA/CLRS-1 filaments can be produced using aerospace bio-packaging materials and regolith powder. After the 3D printing process, the 3D printed PLA/CLRS-1 components were placed in an extraction thimble. Through the reflux of THF, the components slowly dissolved in the solution. The dissolved PLA was then refluxed into a flask along with the THF. The lunar regolith was sieved as needed, which then accumulated in the extraction thimble for collection after the dissolution of the 3D printed PLA–regolith components. Then, the PLA and THF were separated and recycled using the rotary evaporation method, then reserved for future use. The total process lasted for approximately 3 h.

**Figure 11:**
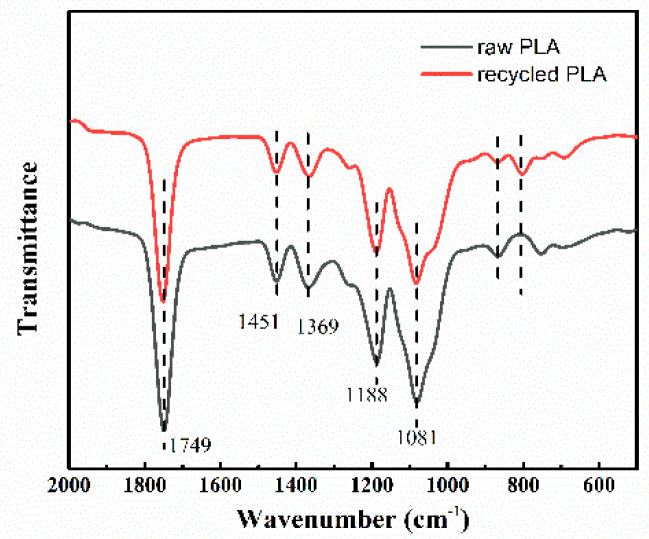
FTIR spectra of raw PLA and recycled PLA.

Next, we analyzed the feasibility of the PLA powder obtained after the recycling process. For this purpose, the chemical structure and molecular weight were analyzed by FTIR-ATR and GPC. Figure 11 presents the FTIR spectra of the raw and recycled PLA samples, which showed the characteristic peak of the C=O group (1749 cm^−1^), methyl group bonds (1451 and 1369 cm^−1^), C–O (1188 cm^−1^), and C–O–C structures (1081 cm^−1^) [41,42]. Therefore, the FTIR spectra preliminary illustrated that no obviously degraded byproducts were found in the recycled PLA, and the recycled PLA could be reused in future applications.

GPC analysis is a useful approach for determining the molecular weights and distributions of polymers. The GPC data of the raw and recycled PLA samples are summarized in Table 6. From Table 6, it is apparent that, compared to raw PLA, the average molecular weight (*M_w_*) of the recycled PLA decreased slightly from 75.85 kg/mol to 73.92 kg/mol. The polydispersity index (*D*) of the recycled PLA also decreased. The *M_w_* value of the recycled PLA was smaller compared to that of the raw PLA, implying the occurrence of compounding-, 3D printing- and recycling-induced molecular weight degradation. Combined with the FTIR results shown above, the decrease in the *M_w_* value was due to the shortening of the PLA molecular chain during the melt extrusion process. 

## 4. Conclusions

In summary, we proposed a simple and effective method for the utilization of lunar regolith resources based on 3D printing, which could potentially be recycled and reused. PLA/CLRS-1 composite filaments were successfully prepared and printed using a material extrusion 3D printing approach. The mechanical properties of the printed composite specimens were slightly reduced upon addition of the lunar regolith. The addition of CLRS-1 to PLA had only a slight influence on the tension and bending strengths of the resulting composites, and they resulted in increased elastic modulus in the composites. SEM and XCT analyses revealed that the increased porosities could cause the lower strengths measured for the composite samples. The addition of lunar regolith also influenced the crystalline process of PLA, shown as the cold crystallization temperature reducing and the corresponding enthalpy improving, while the addition of lunar regolith did not obviously influence the specific crystallinity. Moreover, we successfully recycled the printed PLA/CLRS-1 specimens via the fat extractor reflux method, where GPC and FTIR-ATR tests verified that the recycled PLA had a slightly reduced molecular weight, but was entirely usable for future applications. It is hoped that this study will provide a new perspective for the utilization and recycling of spatial in situ resources such as lunar regolith

## Figures and Tables

**Table 1:** Parameters of 3D printing.

Printing Parameters	Values
Diameter of nozzle	0.4 mm
Nozzle temperature	200 °C
Bed temperature	60 °C
Printing speed	80 mm/s
Layer thickness	0.1 mm
Raster angle	+45°/−45°
Air gap	0.18 mm

**Table 2:** Tensile properties of PLA and PLA/CLRS-1 printed samples.

Samples	σ_max_(MPa)	%R.D.*^a^*	ε_b_ (%)	%R.D.
**Neat PLA**	64.3 ± 3.3	—	7.49	—
**5 wt% CLRS-1**	55.2 ± 2.8	−14.2	6.11	−18.4
**10 wt% CLRS-1**	53.0 ± 2.8	−17.6	4.27	−43.0

*^a^*. percent relative difference.

**Table 3:** Bending properties of PLA and PLA/CLRS-1 printed samples.

Samples	σ_max_(MPa)	%R.D.*^a^*	E(GPa)	%R.D.	ε_b_ (%)	%R.D.
**Neat PLA**	88.0 ± 3.0	—	2.84 ± 0.22	—	—	—
**5 wt% CLRS-1**	86.6 ± 7.0	−1.55	2.96 ± 0.14	4.23	4.87 ± 0.55	—
**10 wt% CLRS-1**	82.7 ± 3.3	−5.98	3.05 ± 0.15	7.39	4.05 ± 0.46	—

*^a^*. percent relative difference.

**Table 4:** The result of porosity analysis via X-ray computed tomography (XCT).

Samples	Porosity (%)	Pore Size		
		Length (mm)	Width (mm)	Pore Spacing (mm)
**Neat PLA**	4.18	0.38	0.37	0.09
**10 wt% PLA/CLRS-1**	5.20	0.33	0.39	0.08

**Table 5:** The thermal properties of 3D printed neat PLA and PLA/CLRS-1 composite specimens.

Samples	*T*_5%_ (°C)	*T*_max_ (°C)	RC^a^ (wt.%)	*T_g_^b^* (°C)	*T_cc_^c^* (°C)	∆*H_cc_^c^* (J/g)	*T_m_^d^* (°C)	∆*H_m_^d^* (J/g)	${\chi_{c}}^{e}$(%)
**Neat PLA**	328	363	0.78	60	123.38	14.76	149.98	17.32	2.75
**5 wt% CLRS-1**	290	344	6.57	61	114.92	22.41	148.49	24.01	1.82
**10 wt% CLRS-1**	279	324	11.95	62	114.09	21.18	148.19	24.08	3.46

^a^ Residual Char (RC) at 700 °C. ^b^ Glass transition temperature (*T_g_*). ^c^ Cold crystallization temperature (*T_cc_*) and enthalpy (*∆H_cc_*). ^d^ Melt temperature (*T_m_*) and enthalpy (*∆H_m_*). ^e^ Crystallinity degree ($\chi_{c}$

).

**Table 6:** Molecular weight variation in PLA before and after recycling.

Samples	*M*_w_ (kg/mol)	D
raw PLA	75.85	1.94
recycled PLA	73.92	1.72
